# Photo‐Switched Catalytic Amyloids

**DOI:** 10.1002/smll.75302

**Published:** 2026-08-18

**Authors:** Shubhra Kanti Bhaumik, Sisira Mambram Kunnath, Elad Arad, Einat Nativ‐Roth, Arunavo Chatterjee, Albert Batushansky, Nitzan Shauloff, Hanna Rapaport, Raz Jelinek

**Affiliations:** ^1^ Department of Chemistry Ben‐Gurion University of the Negev Beer Sheva Israel; ^2^ Ilse Katz Institute (IKI) for Nanoscale Science and Technology Ben‐Gurion University of the Negev Beer Sheva Israel; ^3^ Department of Chemical Engineering Columbia University in the City of New York New York New York USA; ^4^ Avram and Stella Goldstein‐Goren Department of Biotechnology Engineering Ben‐Gurion University of the Negev Beer Sheva Israel

**Keywords:** amyloid fibrils, antibiotic degradation, catalytic amyloids, photo induced catalysis

## Abstract

Light–mediated assembly of catalytic agents furnishes powerful means for spatiotemporal control of chemical reactions. Here, we demonstrate photo‐switched hierarchical assembly of catalytic amyloid fibrils, through mixing an amphiphilic cross‐ß peptide with a merocyanine photo‐switch. The peptide monomers, comprising phenylalanine‐lysine repeats, do not self‐assemble in water because of the electrostatic repulsion between the positively charged lysine side chains; however, upon light‐induced merocyanine‐spiropyran transformation, amyloid fibrils were formed. Interestingly, increasing the mole ratio between the merocyanine photo‐switch and the peptide gave rise to a remarkable assembly of aligned elongated fibrils. The process was fully reversible as light turn‐off resulted in fibril disintegration. Notably, the photoinduced amyloid fibrils catalyzed hydrolysis of β‐lactam antibiotics, offering a platform for light‐mediated degradation of antibiotic pollutants in water. Microscopic and spectroscopic experiments reveal that the photoinduced transient catalytic amyloids were likely formed through π–π interactions between the spiropyran moieties and the aromatic residues of the phenylalanines, while electrostatic interactions between the negative sulfonates and lysine sidechains on the fibrils’ surface were responsible for the macroscale alignment. The amyloid‐merocyanine system represents a new concept for light‐regulated assembly of catalytic amyloids, which may have also played a role in prebiotic evolution processes.

## Graphical abstract

Photo‐switched reversible hierarchical assembly of catalytic amyloid fibrils is accomplished by mixing an amphiphilic cross‐ß peptide with a merocyanine photo switch. The peptide monomers, comprising phenylalanine‐lysine repeats, do not self‐assemble in water because of the electrostatic repulsion. However, upon light‐induced merocyanine‐spiropyran transformation, amyloid fibrils are formed. The amyloids catalyse hydrolysis of water‐soluble antibiotics, furnishing light‐regulated catalysis.

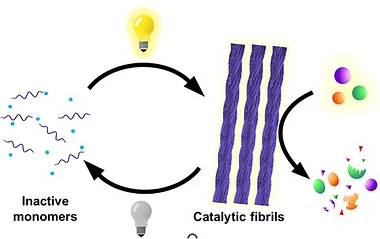

## 1 Introduction

Catalytic amyloids [1, 2, 3, 4], both synthetic and naturally occurring [5, 6, 7, 8, 9], have garnered considerable attention in recent years, underscoring new avenues in amyloid pathologies, biochemical processes, and prebiotic chemistry [10, 11, 12, 13, 14, 15]. Indeed, fibrillar peptide organizations in amyloid systems have been shown to provide templates for catalytic sites pertaining to diverse reactions [16, 17, 18, 19, 20, 21, 22, 23, 24, 25, 26, 27]. A recent groundbreaking study demonstrated that functional bacterial amyloids and their synthetic biomimetic peptide counterparts catalyze hydrolysis of β‐lactam antibiotics [28, 29], pointing to both unrecognized mechanisms for bacterial drug resistance, as well as amyloids as a potential platform for tackling antibiotic contamination in water, a growing health and environmental hazard [30, 31].

Photo‐regulated catalysis may be a particularly potent strategy for manipulating chemical reactions as light can be controlled spatially and temporally, and also tunable in both wavelength and intensity. Photo‐modulated catalysis has been primarily accomplished utilizing photo‐switchable molecules covalently attached to organic or organometallic catalysts [32, 33, 34]. Chemical reactivity of such systems is generally controlled through structural photo‐switch [32, 33, 34]. Recent studies reported light‐mediated peptide assemblies with catalytic properties utilizing synthetic modification of the peptides with photo‐switchable moieties [35, 36, 37]. Zhao et al. [37] and Samanta et al. [36] reported photoinduced esterase activity in azobenzene‐functionalized synthetic peptides, occurring through *cis‐trans* transformations of the azobenzene moieties. In a similar concept, Zhang and co‐workers reported photo‐induced catalysis employing a synthetically modified merocyanine‐based short peptide [35]. These systems, however, require multistep synthesis for constructing the peptide/photo‐responsive composites. Moreover, optical switching in these systems is either carried out by dual wavelength light irradiation [e.g., ultraviolet (UV) and visible], or catalysis, in fact, is turned off under light, overall limiting potential applicability.

Light‐regulated assembly/disassembly of amyloids has rarely been achieved. Xuan et al. reported photo‐reversible amyloid nano‐neutrophil extracellular traps (nanoNETs) in which the amyloid aggregates disassembled upon near infrared (NIR) irradiation. Interestingly, that study demonstrated reversible lysozyme amyloid flexible nanofibrils, enabling pathogen elimination and tissue regeneration [38]. Here, we demonstrate a photo‐switched hierarchical assembly of catalytic amyloids. Light irradiation plays a dual functional role – both triggering amyloid fibril formation and spatial organization, as well as inducing catalysis. The photo‐switched catalytic amyloid platform comprised of a mixture of a merocyanine molecule (transformed upon irradiation with white light to a spiropyran) and an amyloid‐forming peptide comprising alternating phenylalanine‐lysine residues. We demonstrate that fibril formation occurred exclusively upon light irradiation, arising from the combined effect of hydrophobic interactions between the light‐transformed spiropyran and phenylalanine residues of the peptides, and electrostatic attraction between the spiropyran‐linked alkyl sulfonate residues and pendant lysine sidechains of the peptide. Notably, the photo‐switched amyloid fibrils displayed a remarkable liquid crystalline organization, likely afforded by the light‐mediated attenuation of the electrostatic repulsion between the lysine sidechains displayed upon the fibrils’ surface. Moreover, the light‐triggered amyloid fibrils exhibited excellent catalytic activity toward β‐lactam hydrolysis, underscoring an innovative concept of light‐regulated reversible functional amyloids.

## 2 Results and Discussion

### 2.1 Design Principles of the Optically Switched Catalytic Amyloids

Figure 1 depicts the optically switched catalytic amyloid concept. We employed an amyloidogenic cross‐ß peptide exhibiting an alternating lysine/phenylalanine motif capped by proline residues with the sequence PKFKFKFKFKFKP (denoted PFK) (Figure 1A). PFK does not assemble into amyloid fibrils in water because of the pronounced electrostatic repulsion between the positively charged lysine sidechains [39, 40, 41]. Surprisingly, however, when the merocyanine photo switch (E)‐3‐(2‐(2‐hydroxystyryl)‐3,3‐dimethyl‐3H‐indol‐1‐ium‐1‐yl)propane‐1‐sulfonate (denoted **MCH**, Figure 1B) [42] was added to the aqueous solution of PFK and irradiated with white light, remarkable photoinduced assembly of transient amyloid fibrils occurred (Figure 1C). Specifically, upon light irradiation, the photoinduced transformation of **MCH** to the spiropyran (**SP**) (Figure 1B) gave rise to the formation of elongated fibrils exhibiting liquid crystalline‐like alignment (Figure 1C). Upon turning off illumination, the fibrils disintegrated, reverting to the initial monomeric state (Figure 1C). Interestingly, the photoinduced PFK/**SC** amyloid fibrils further exhibit catalytic activity, accelerating hydrolysis of ß‐lactam antibiotics [29] (Figure 1C).

**FIGURE 1:**
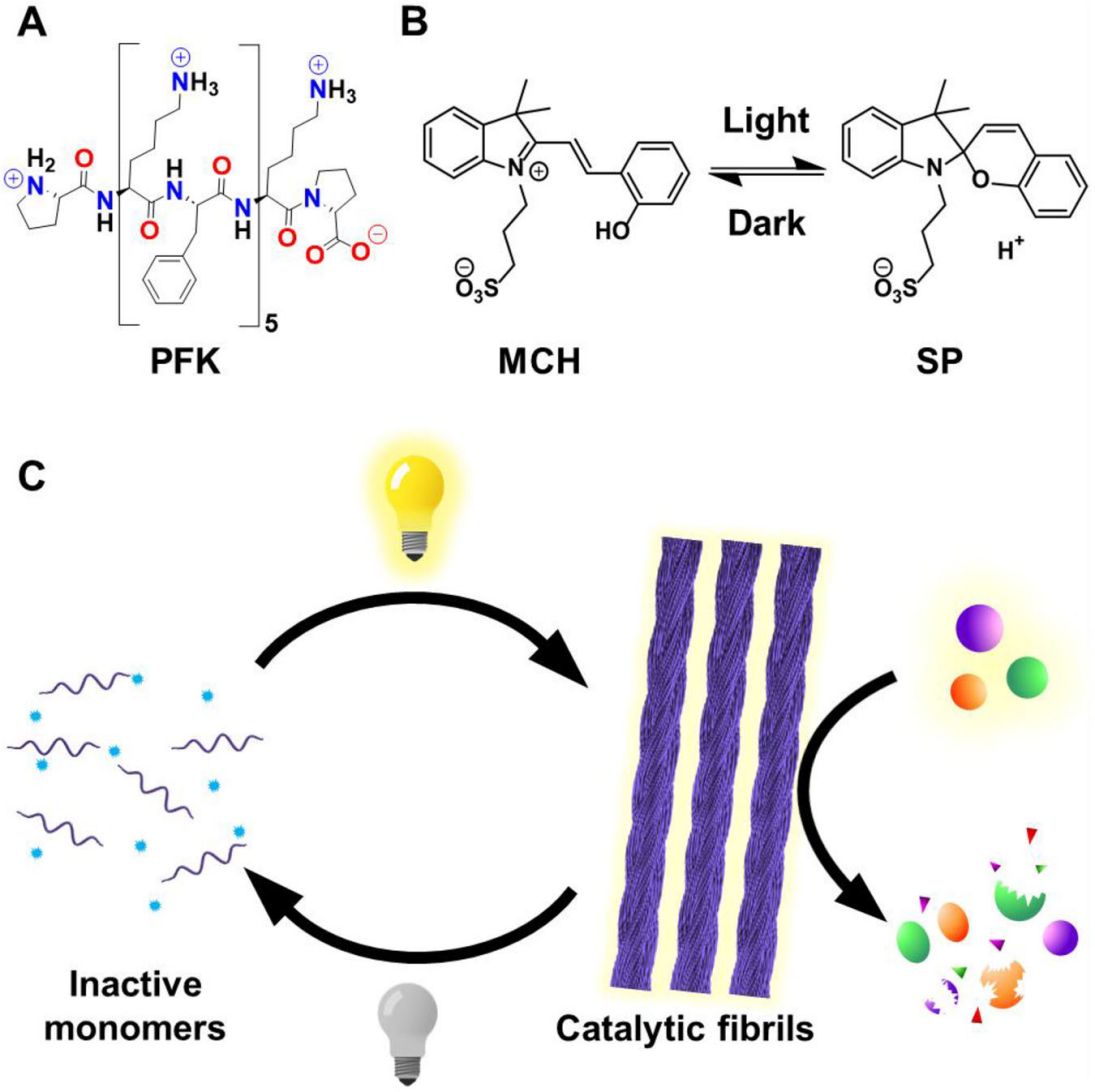
Optical switching of catalytic amyloid fibrils comprising the amphiphilic cross‐**ß** peptide PFK and the merocyanine photo‐switch. (A) Chemical structure of PFK comprising alternating Phe‐Lys repeats and capped by proline residues. (B) The photoinduced transition of the merocyanine **MCH** to the spiropyran **SP**. (C) The experimental concept: an aqueous mixture of PFK (twisted string) and **MCH** (blue spheres) is transformed into aligned amyloid fibrils upon light irradiation. The fibrils exhibit catalytic properties (represented by degradation of the colored spheres). The process is reversible as the fibrils revert to the initial monomeric species upon light turnoff.

**FIGURE 2:**
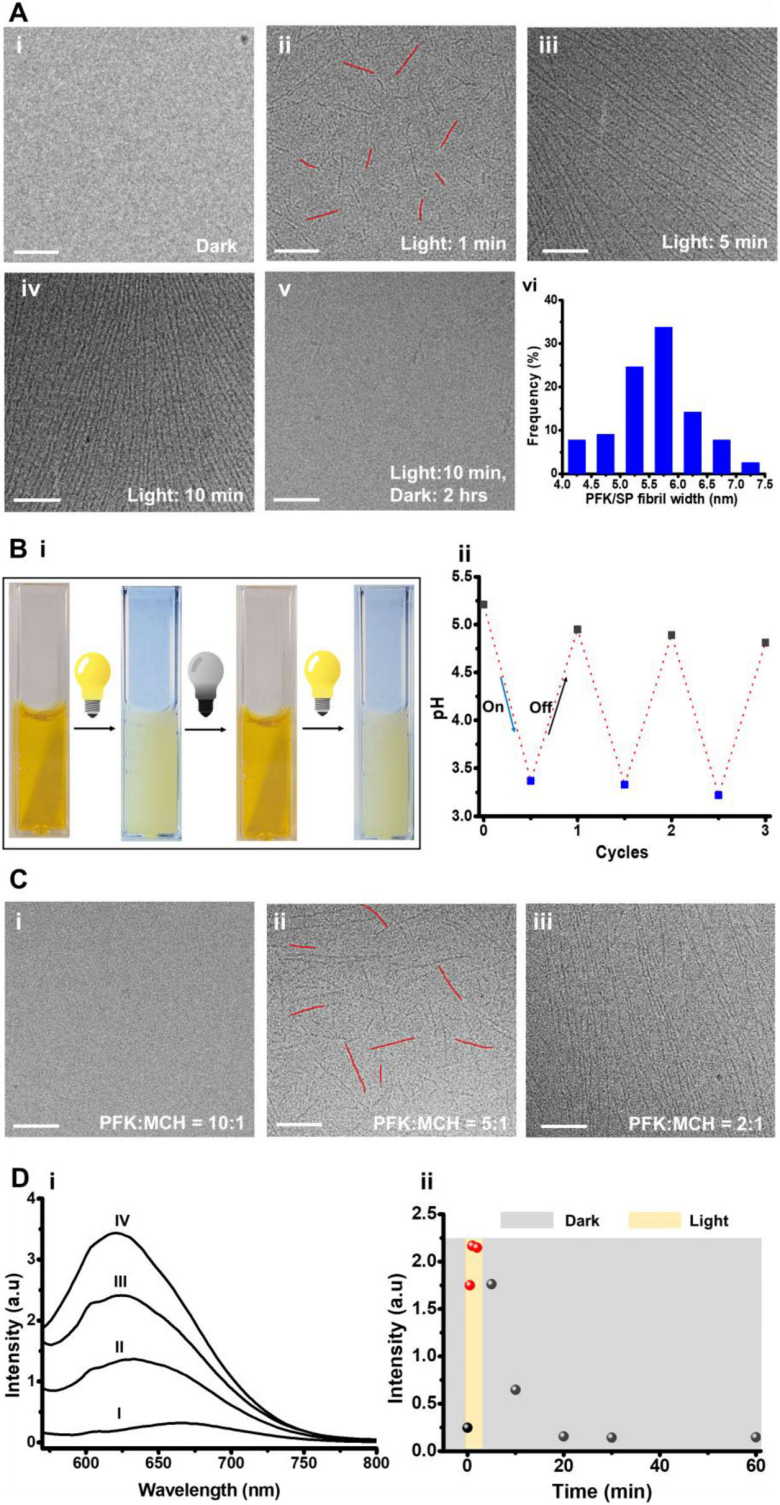
Characterization of the photoinduced fibril formation in the PFK/**MCH** mixture. (A) Cryo‐TEM images of PFK (0.5 mM)/**MCH** (1.0 mM). (i) initial, dark condition; (ii) under 1 min. white light irradiation (the fibrils are highlighted in red to better visualize), (iii) 5 min illumination, (iv) 10 min illumination; (v) 2 h after turning off light irradiation. Scale bars: 100 nm. (vi) Thickness distribution of the fibrils, determined from the cryo‐TEM analysis. (B) (i) Photographs of the PFK/**MCH** mixture (1:2 mole ratio) without and with illumination in two repetitions. (ii) pH of the PFK/**MCH** mixture (1:2 mole ratio) in dark and light irradiation upon several cycles. The arrows indicate light on and off, respectively. (C) Cryo‐TEM images of PFK/**MCH** mixtures having different mole ratios between PFK and **MCH**, following 10‐min white light irradiation. (D) (i) Fluorescence spectra of Amytracker‐680 (λ_exc_ = 550 nm) of PFK (0.5 mM)/**MCH** at different PFK:**MCH** mole ratios under white light irradiation (10 min). I: 5:1, II: 1:1, III: 1:1.6, and IV: 1:2 mole ratios. (ii) Amytracker‐680 (λ_ext_ = 550 nm) fluorescence emission (at 620 nm) vs time under dark and white light illumination, PFK and **MCH** concentrations were 0.5 and 1.0 mM, respectively.

### 2.2 Structural and Mechanistic Analyses of the Optically Switched Fibril Assembly

Microscopic and spectroscopic analyses of the reversible light‐triggered amyloid assembly are presented in Figure 2. Cryogenic transmission electron microscopy (cryo‐TEM) imaging in Figure 2A demonstrates the direct relationship between fibril assembly in the PFK/**MCH** solution (1:2 mole ratio between PFK and **MCH**) and the extent of light irradiation. Prior to illumination, no fibrils formed (Figure 2A, i). However, upon 1‐min irradiation (white light, 380–750 nm range, power of 70 mW cm^−2^), ubiquitous short fibrils (50–200 nm) formed (Figure 2A, ii). Remarkably, longer irradiation times (5 and 10 min) induced the assembly of very long (1‐2 microns) aligned fibrils (Figure 2A‐iii, iv). Similar nematic phase‐like fibril organization was observed in high salt, order‐of‐magnitude higher concentration peptide solutions, following incubation for several days [43]. Polarized optical microscopy (POM) analysis of the light‐irradiated PFK/**MCH** co‐mixture further supports the formation of a nematic phase‐like fibril organization (Figure S1).

Upon turning off illumination, complete fibril disintegration occurred, underscoring the reversibility and transient nature of the photoinduced amyloid fibril formation (Figure 2A‐v). The widths of the photo‐induced PFK/**SP** fibrils were relatively uniform, as reflected in the bar diagram in Figure 2A‐vi, yielding an average thickness of 5.6 ± 0.7 nm. Interestingly, this value is higher than the thickness of bare PFK fibrils [39] and salt‐induced PFK fibrils (Figure S2) [29] likely indicating the presence of PFK and **SP** within the fibrils.

Fibril assembly was also visible to the naked eye as the solution containing PFK/**MCH** turned opaque under light irradiation due to the formation of the PFK/**SP** fibrils (Figure 2B‐i). Echoing the cryo‐TEM results in Figure 2A, the solution appearance transformation was reversible upon light turn‐off. Notably, the pH of the PFK/**MCH** aqueous solution could be similarly cycled upon light on/off repetitions, decreasing from ∼5.2 to ∼3.3 upon light irradiation (Figure 2B‐ii). This pH change accounts for the reversible photoinduced transformation of **MCH** into **SP**, which is acidic (i.e., Figure 1B), upon light irradiation [42].

Figure 2C presents cryo‐TEM images of the PFK/**MCH** mixture at different mole ratios between the two species, irradiated for 10 min with white light. Indeed, Figure 2C demonstrates that the presence of both **MCH** and PFK is essential both for initiating amyloid fibril assembly, as well as affecting macroscale fibril organization. Specifically, no fibrillation occurred when light irradiation was applied to a solution comprising PFK and **MCH** at a mole ratio of 10:1 (Figure 2C,i). Randomly oriented short fibrils formed upon light irradiation of a solution comprising higher abundance of **MCH** (PFK/**MCH** ratio 5:1; Figure 2C‐ii). Figure 2C‐iii reveals that an assembly of long, aligned fibrils occurred upon illuminating a solution in which the PFK:**MCH** ratio was further decreased to 2:1. The microscopy data in Figure 2C attest to the key role of **SP** in fibril formation and organization.

The optically‐switched amyloid fibrillation process was also studied by monitoring the fluorescence intensity of Amytracker‐680, which is correlated with amyloid fibril assembly [44] (Figure 2D). In the experiments, the fluorescent dye was added to the PFK/**SP** fibrils under light irradiation (10 min) at different PFK:**SP** ratios (Figure 2D‐i; PFK concentration = 0.5 mM), and at different dark/light durations (Figure 2D‐ii). Echoing the cryo‐TEM data in Figure 2C, the spectra in Figure 2D‐i show that the Amytracker‐680 fluorescence significantly increased in tandem with the PFK:**MCH** ratio, confirming that the photoinduced **SP** photoacid was the key determinant for fibril formation. Amytracker‐680 fluorescence emission analysis also underscores the light‐induced fibrillation kinetics (Figure 2D‐ii). Specifically, fibril assembly occurred within 2 min of light irradiation, giving rise to a significant increase in fluorescence emission of the dye (Figure 2D‐ii). Notably, the Amytracker‐680 fluorescence reverted to the initial low (pre‐fibril) intensity value approximately 20 min after turning off illumination, attesting to the reversibility of the photoinduced PFK/**SP** amyloid fibril formation.

Physicochemical analyses in Figure 3 shed light on the structural and mechanistic features of the optically switched amyloid fibril assembly. Figure 3A depicts zeta potential measurements in the PFK/**MCH** mixture (1:2 mole ratio) under dark and upon light irradiation. An initial zeta potential value of ∼7 mV likely accounts for small non‐fibrillar assemblies in the non‐irradiated PFK/**MCH** mixture. Upon light irradiation, zeta potential of 15 mV was recorded and persisted throughout the entire illumination duration. The higher zeta potential is ascribed to the organized fibril assembly, surface‐displaying ubiquitous positive lysine sidechains. Notably, after light turnoff, the zeta potential reverted within ∼40 min to the initial value (prior to light illumination), reflecting the gradual fibril disintegration and reversibility of the photoinduced amyloid formation process (i.e., Figure 2A).

**FIGURE 3:**
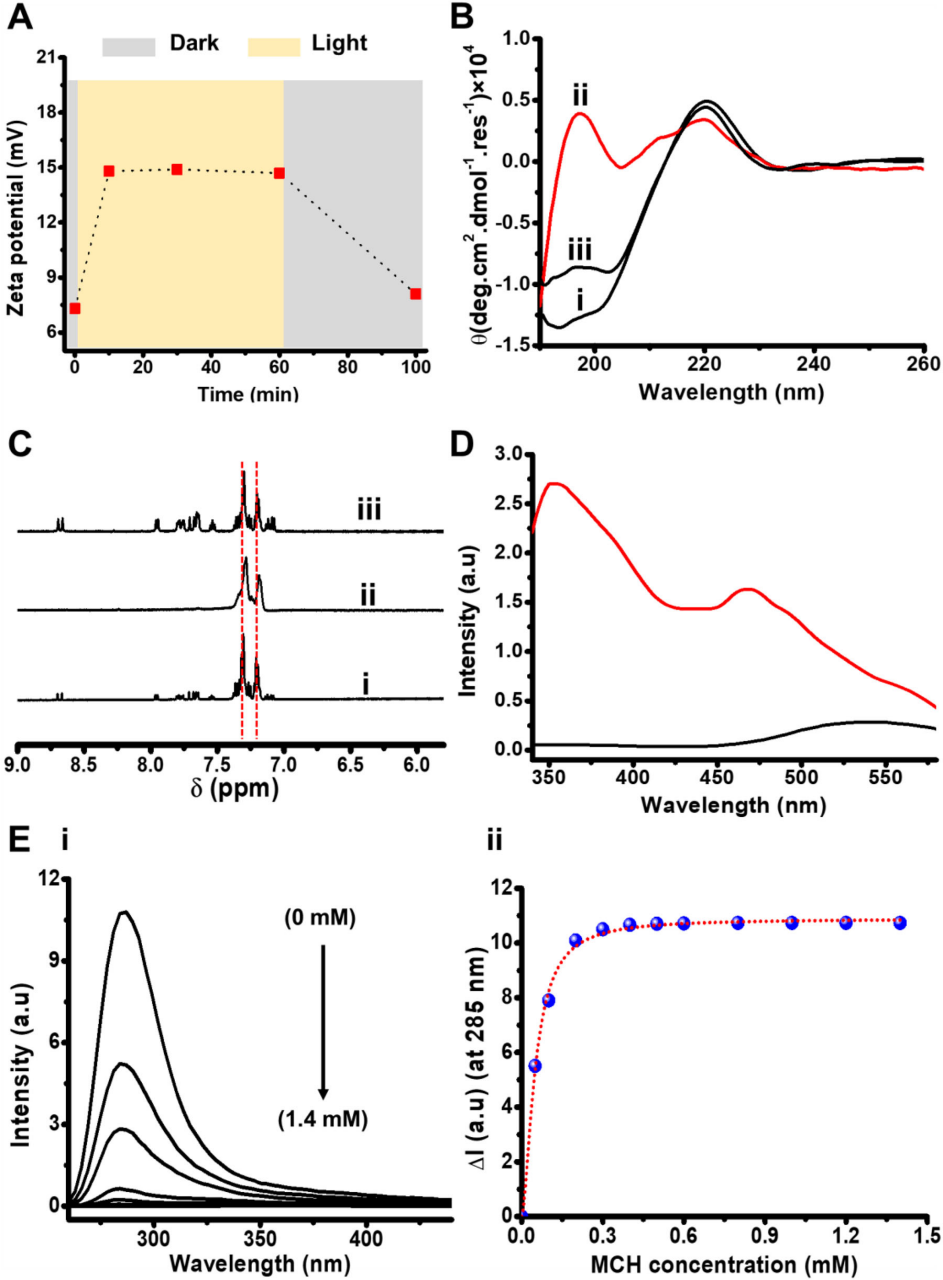
Molecular determinants of the optically switched PFK/**SP** fibril assembly. (A) Zeta potential (ζ) recorded in the PFK (0.5 mM)/**MCH** (1.0 mM) mixture under dark and upon white light illumination. (B) CD spectra, and (C) ^1^H NMR spectra in D_2_O (aromatic region: 5.8–9 ppm; red dotted lines indicate ^1^H peaks from the phenylalanine residues in PFK). (i) Dark conditions, (ii) upon white light illumination, (iii) dark conditions, 20 min after light irradiation. (D) Fluorescence emission spectra (λ_ex_ **_c_** = 300 nm). Red spectrum: PFK/**SP** under light (PFK concentration was 0.5 mM and **MCH** concentration was 1.0 mM). Black spectrum: **SP** alone (no PFK present) [generated in a **MCH** (1.0 mM) solution irradiated with white light for 10 min]. (E) (i) PFK autofluorescence spectra (λ_ext_ = 240 nm) following addition of **MCH** (0 to 1.4 mM) and irradiation with white light for 10 min. (ii) Hill plot depicting the change in PFK autofluorescence intensity in the PFK**/MCH** mixtures upon illumination with white light for 10 min.

Circular dichroism (CD) spectra recorded in the PFK/**MCH** mixture (1:2 mole ratio) shed light on the structural features of the photoinduced fibrils (Figure 3B). In the initial, dark condition, the CD spectrum features a minimum at 198 nm corresponding to random coil, while the minimum at 205 nm with a broad positive peak at around 220 nm (Figure 3B‐i), ascribed to a polyproline‐type II conformation of PFK monomers [45, 46]. A pronounced structural transformation of PFK occurred upon illumination, yielding a broad positive peak at 195 nm and a shallow positive signal at >205 nm, likely reflecting the superposition of multiple conformational contributions including π–π stacking [47] and a shallow dip at around 214 nm, indicating an anti‐parallel ß‐sheet conformation (Figure 3B‐ii) [29, 41, 48]. Notably, the CD spectrum recorded 20 min after turning off light irradiation reverts to the initial state (Figure 3B‐iii), echoing the TEM analysis (Figure 2) and zeta potential measurements (Figure 3A), indicating fibril disintegration. Attenuated Total Reflectance Fourier‐Transform Infrared (ATR‐FTIR) experiments echo the CD results, underscoring the reversible photoinduced PFK structural transformation (Figure S3).

Spectroscopic experiments presented in Figure 3C–E illuminate the interactions between PFK and **MCH** (transformed into **SP** upon illumination) in the amyloid fibrils. Figure 3C depicts ^1^H NMR spectra in the aromatic region, confirming significant interactions between **MCH** and PFK, upon light irradiation. The NMR spectrum of PFK/**MCH** in Figure 3C‐i reveals sharp peaks corresponding to the aromatic protons of **MCH**, as well as two major signals at ∼7.2 and ∼7.3 ppm (red dotted lines) assigned to the phenyl residues of PFK. In comparison, the ^1^H NMR spectrum of the PFK/**MCH** mixture upon light irradiation in Figure 3C‐ii depicts only the two PFK peaks (the spectrum was acquired in situ using a 405 nm optical fiber inserted into the NMR tube). The disappearance of the **MCH** ^1^H signals under light irradiation attests to binding between the photoinduced **SP** molecules and PFK. Importantly, the ^1^H spectrum reverted to the initial state after turning off illumination (Figure 3C‐iii), reflecting the reversibility and transient nature of the photoinduced fibril formation.

Fluorescence spectroscopy measurements in Figure 3D furnish additional information on the interactions between the optically switched **SP** and PFK. The fluorescence emission spectrum of **SP** alone (excitation at 300 nm), induced upon white light illumination of 1.0 mM **MCH** solution, reveals low fluorescence at ∼550 nm, corresponding to residual **MCH** molecules (Figure 3D; the fluorescence spectrum of **MCH** is shown in Figure S4). Interestingly, significantly higher fluorescence emission was recorded in the photoinduced **SP/**PFK mixture, with peaks appearing at around 360 and 475 nm. The fluorescence peak at 360 nm likely accounts for the restricted motion of **SP** molecules, incorporated within the fibrils [49, 50], while the signal at 475 nm likely corresponds to internal charge transfer between amide bonds and lysine residues within PFK [51, 52, 53].

Phenylalanine autofluorescence analysis in Figure 3E further attests to pronounced interactions between the photoinduced **SP** molecules and Phe residues of PFK. Specifically, Figure 3E,i demonstrates that the fluorescence peak at 285 nm (excitation at 240 nm), corresponding to the emission of phenylalanine residues in PFK [54, 55], decreased significantly upon addition of **MCH** and concomitant light irradiation. The Hill plot in Figure 3E‐ii, depicting the change in fluorescence emission intensity vs **MCH** concentration, yielded a **SP**‐PFK binding constant of ∼2 × 10^4^ M^−1^, indicating moderate binding between **SP** and PFK [56]. Overall, the autofluorescence results in Figure 3E suggest that the affinity of **SP** to the Phe residues in PFK, likely due to π–π interactions between the aromatic units of the two species, constitutes a key determinant in the photoinduced **SP/**PFK fibril assembly.

To further investigate the contributions of specific moieties in **SP** to fibril formation and macroscale organization, we tested the assembly properties of PFK and two **SP‐**related molecules (Figure 4). Specifically, Figure 4A depicts cryo‐TEM imaging and PFK autofluorescence spectra recorded for an aqueous solution of PFK and 1',3',3'‐trimethylspiro[chromene‐2,2'‐indoline] (denoted **SP_c_**), which exhibits the same spiropyran core as **SP**, but lacks the charged n‐propyl sulfonate sidechain. The cryo‐TEM image in Figure 4A‐i reveals that fibrils were formed upon incubating **SP_c_** with PFK (PFK: 0.5 mM, **SP_c_**: 1.0 mM). Notably, though, the PFK/**SP_c_** fibrils were short and randomly distributed, significantly different than the long, aligned fibril arrays observed in the case of photoinduced PFK/**MCH** (at the same concentrations as the PFK/**SPc;** e.g., Figure 2A,B). CD (Figure S5A) and FTIR (Figure S5B) data indicate that the PFK/**SP_c_** fibrils adopted anti‐parallel ß‐sheet conformations, like the PFK/**MCH** systems. Autofluorescence spectra of PFK in the presence of **SP_c_** in Figure 4A‐ii demonstrate quenching of PFK fluorescence emission, echoing the results obtained for the PFK/**MCH** solutions (i.e., Figure 3E), suggesting predominance of π–π interaction between the spiropyran core of **SP_c_** and Phe residues of PFK. ^1^H NMR spectroscopy experiments in Figure S6 confirm the occurrence of binding between **SP_c_** and PFK.

**FIGURE 4:**
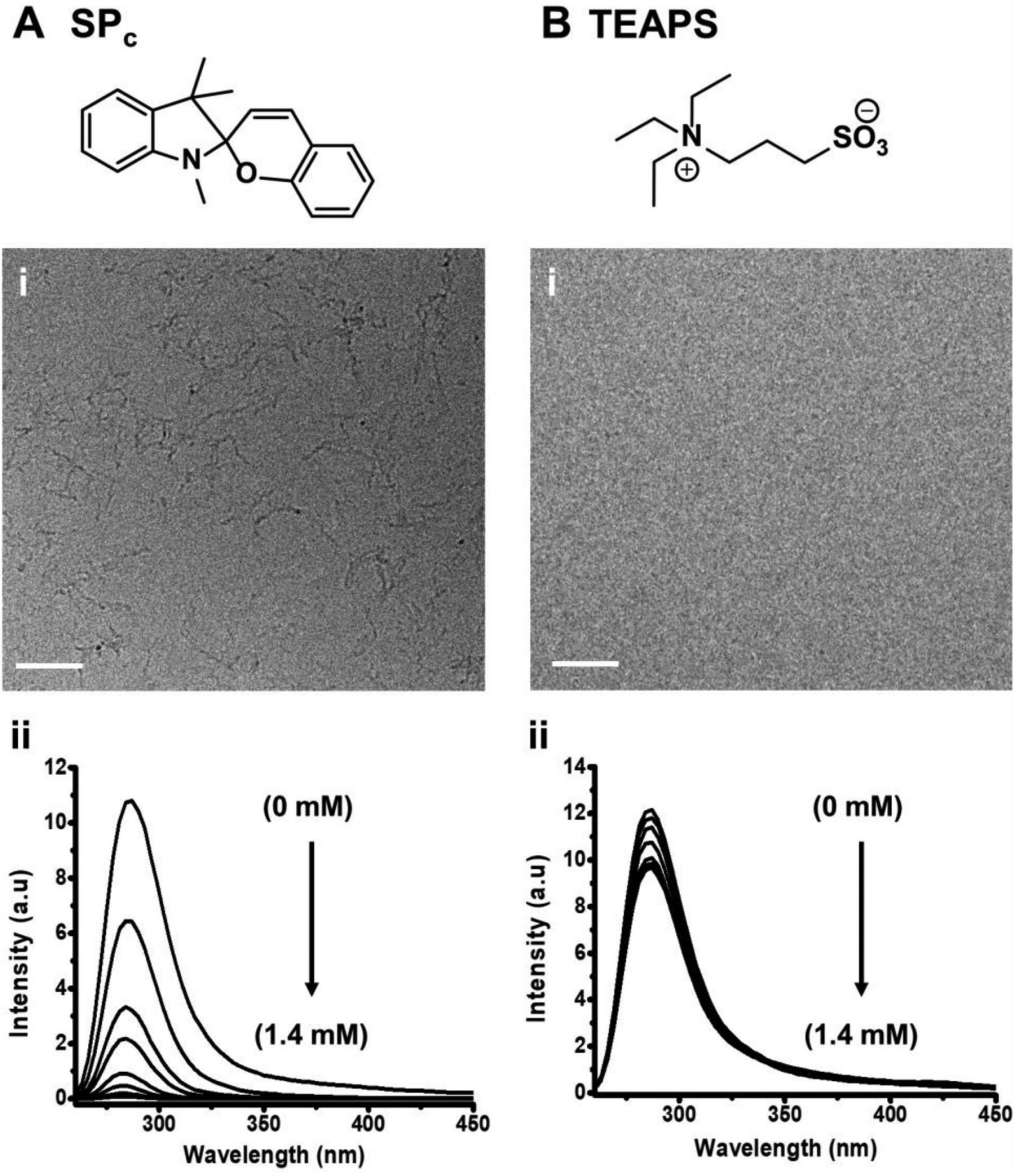
Assembly properties and bimolecular interactions of PFK with small molecules comprising units of photoinduced **SP**. A. The spiropyran derivative **SP**_**c**_ (molecular structure shown above): (i) A Cryo‐TEM image of PFK (0.5 mM)/**SP**_**c**_ (1.0 mM). Scale bar: 100 nm. (ii) Autofluorescence spectra of PFK (0.5 mM) (λ_exc_ = 240 nm) upon addition of different concentrations of **SP**_**c**_. B. The n‐propyl‐sulfonate derivative **TEAPS** (molecular structure shown): (i) A Cryo‐TEM image of PFK (0.5 mM)/**TEAPS** (1.0 mM). Scale bar: 100 nm. (ii) Autofluorescence spectra of PFK (0.5 mM) (λ_exc_ = 240 nm) upon addition of different concentrations of **TEAPS**.

Yet in another set of experiments, presented in Figure 4B, we assessed the assembly properties of PFK incubated with 3‐(triethylammonio)propane‐1‐sulfonate (denoted as **TEAPS**), which comprises of the same sulfonate sidechain as **SP**, attached to a quaternary ammonium moiety rather than the spiropyran residue as in **SP**. Importantly, **TEAPS** did not give rise to fibril formation upon mixing with PFK, as evident from the cryo‐TEM image in Figure 4B‐i and the minute decrease in the PFK autofluorescence (Figure 4B‐ii). CD analysis similarly indicated no fibril assembly in the PFK/**TEAPS** mixture (Figure S7).

The experiments in Figures 2, 3, 4 underscore the key roles of the spiropyran core of **SP** in **SP/**PFK fibril formation, and the contribution of the n‐propyl‐sulfonate moieties as a structural determinant for subsequent alignment of the photoinduced PFK/**SP** fibrils. Figure 5 presents further experimental data and a proposed structural model for the optically switched amyloid fibril assembly process. Figure 5A depicts a comparative analysis of the Amytracker‐680 fluorescence evolution in PFK/**SP_c_** and PFK/**SP** mixtures, furnishing insight into the optically switched fibrillation process.

**FIGURE 5:**
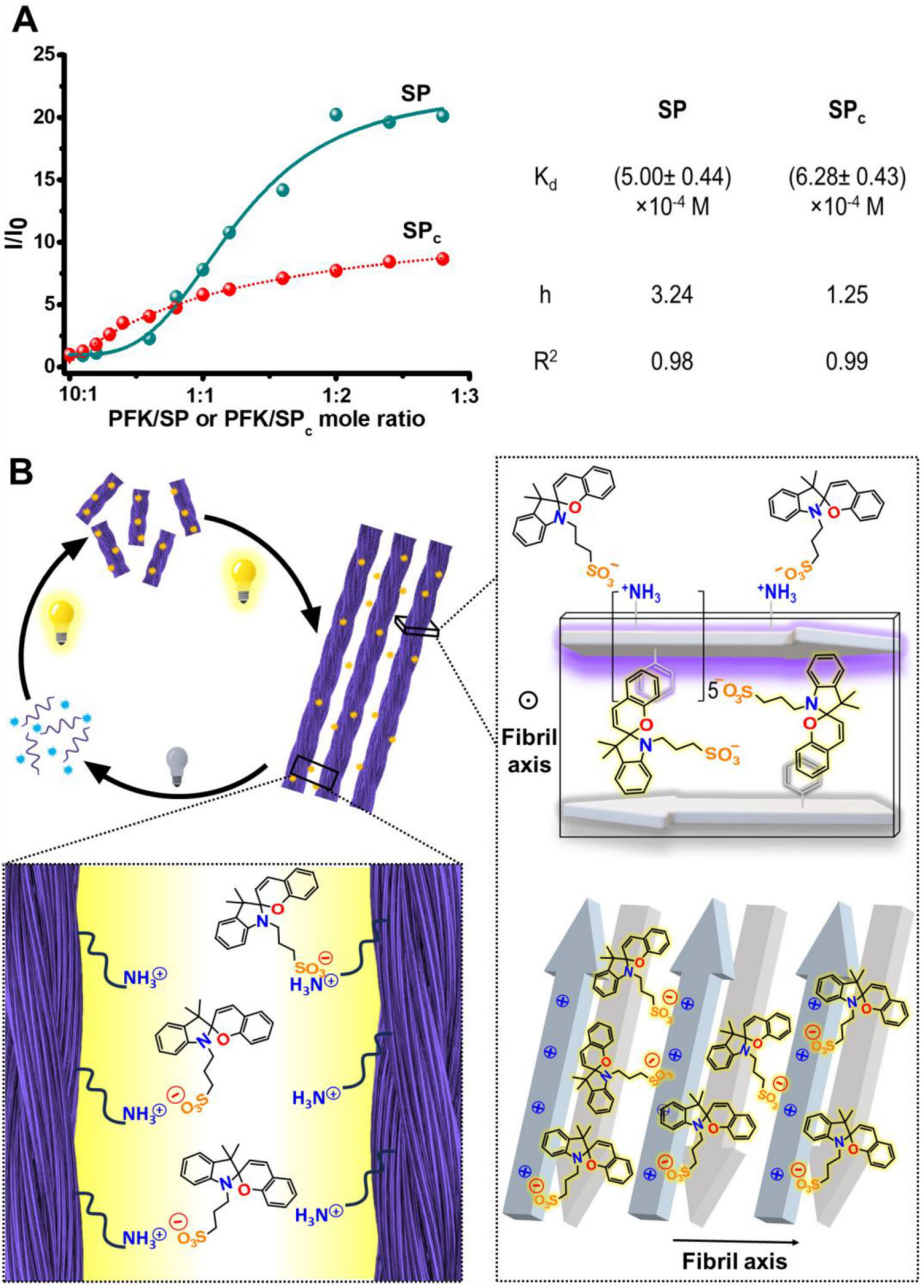
Light‐induced fibrillation in the PFK/**MCH** mixture. (A) Amytracker‐680 fluorescence emission intensity (I/I_0_ – fluorescence intensity over initial fluorescence intensity) recorded vs mole ratios between PFK and **SP** or **SP_c_** upon white light illumination (10 min) (left). Hill parameters extracted from fitting the two curves are presented on the right. (B) Proposed structural model for the optically switched hierarchical amyloid fibril assembly. PFK monomers (twisted strings) mixed with **MCH** (blue spheres) are transformed into short random fibrils upon illumination, further forming aligned assembly of elongated PFK/**SP** amyloid fibrils. The process is reversible and the aligned fibrils disintegrate upon light turnoff. The π‐π interactions between the spiropyran units of **SP** and the aromatic residues of phenylalanine, and electrostatic attraction between the sulfonate residues of **SP** and lysine sidechains, which together account for the light‐induced fibril assembly, are indicated.

Notably, while the fluorescence intensity (and concomitant fibril assembly) in the PFK/**SP_c_** system appears to exhibit a hyperbolic trend upon increasing concentration, the fibrillation process in the case of photoinduced PFK/**SP** displays a clear sigmoidal curve (Figure 5A). Hill‐Langmuir fit of the fluorescence curves recorded for PFK/**SP** and PFK/**SP_c_** display dissociation constant (*k_d_*) values of ∼6.3 × 10^−4^ and ∼5.0 × 10^−4^ M, respectively (Figure 5A, right). The calculated Hill coefficient (*h*) for PFK/**SP** is 3.24, which is significantly smaller for PFK/**SP_c_** (1.25, Figure 5A right), indicating that the photo‐induced PFK/**SP** fibrillation is allosteric, accounting for the sigmoidal Amytracker‐680 fluorescence curve (Figure 5A).

Indeed, the difference in the Amytracker‐680 fluorescence emission kinetics in Figure 5A reflects distinct fibrillation pathways. For PFK/**SP_c_**, the hyperbolic nature of the fibrillation isotherm likely attests to progressive concentration‐dependent fibril growth, promoted by hydrophobic/π–π interactions between the spiropyran core of **SP_c_** and phenylalanine residues of PFK. In comparison, the sigmoidal fibrillation curve in the optically switched PFK/**MCH** mixture likely reflects a two‐step process schematically depicted in Figure 5B. Essentially, photoinduced transformation of **MCH** to **SP** accounts for an initial assembly process yielding short, randomly dispersed fibrils (Figure 5B) stabilized by both π–π interactions between spiropyran and the aromatic units of phenylalanine and electrostatic interactions between the lysine sidechains and sulfonate residues of **SP**. This step is followed by fibril elongation and alignment, facilitated by electrostatic affinity between the positive lysine sidechains on the fibrils’ surface and the negative sulfonate residues. Indeed, fibril elongation and alignment occur in solutions comprising high **MCH**:PFK mole ratios and upon long illumination (i.e., cryo‐TEM images in Figure 1). At a very low **MCH** concentration, e.g., a PFK:**MCH** ratio of 10:1, the concentration of photo‐transformed **SP** is insufficient to compensate for the electrostatic repulsion between the peptides, giving rise to the cooperative fibril assembly. Due to significantly high cooperativity (*h* = 3.24, Figure 5A), isolated **SP** molecules are unable to generate **SP**‐mediated ordered packing with PFK to form fibrils. Notably, as shown in Figure 5B, the fibrillation process is reversible, reverting to the initial monomeric state upon light turn‐off.

### 2.3 Hydrolysis of ß‐Lactam Antibiotics by Optically Switched Amyloid Fibrils

Amyloids exhibiting biocatalytic properties have been recently reported, including metal nanoparticles‐decorated ß‐lactoglobulin amyloid fibril‐based membranes for continuous flow catalysis [57], and iron‐anchored amyloid hydrogels for selective conversion of alcohols into acetic acid [58]. We used the photo‐switched amyloid assembly concept to achieve light‐triggered functionality, specifically, the hydrolysis of water‐soluble antibiotic compounds (Figure 6). Specifically, Figure 6 demonstrates that the optically switched PFK/**SP** amyloid fibrils exhibited remarkable photoinduced catalytic activity, accelerating hydrolysis of ß‐lactam antibiotics. Figure 6A shows that the optically switched PFK/**MCH** amyloid fibrils catalyzed the hydrolysis of the chromogenic antibiotic nitrocefin, a widely used ß‐lactam surrogate [59]. Figure 6A,i presents ultraviolet‐visible (UV–vis) absorbance spectra of nitrocefin (2 mM) added to an aqueous solution of PFK/**MCH** (1:2 mole ratio), which was subsequently illuminated by white light for different durations. The UV–vis absorbance spectra in Figure 6A,i reveal light‐irradiation‐dependent decrease in the intensity of the peak at 400 nm, assigned to non‐hydrolyzed nitrocefin [60], accompanied by an increase in the signal at 492 nm, corresponding to hydrolyzed nitrocefin.

**FIGURE 6:**
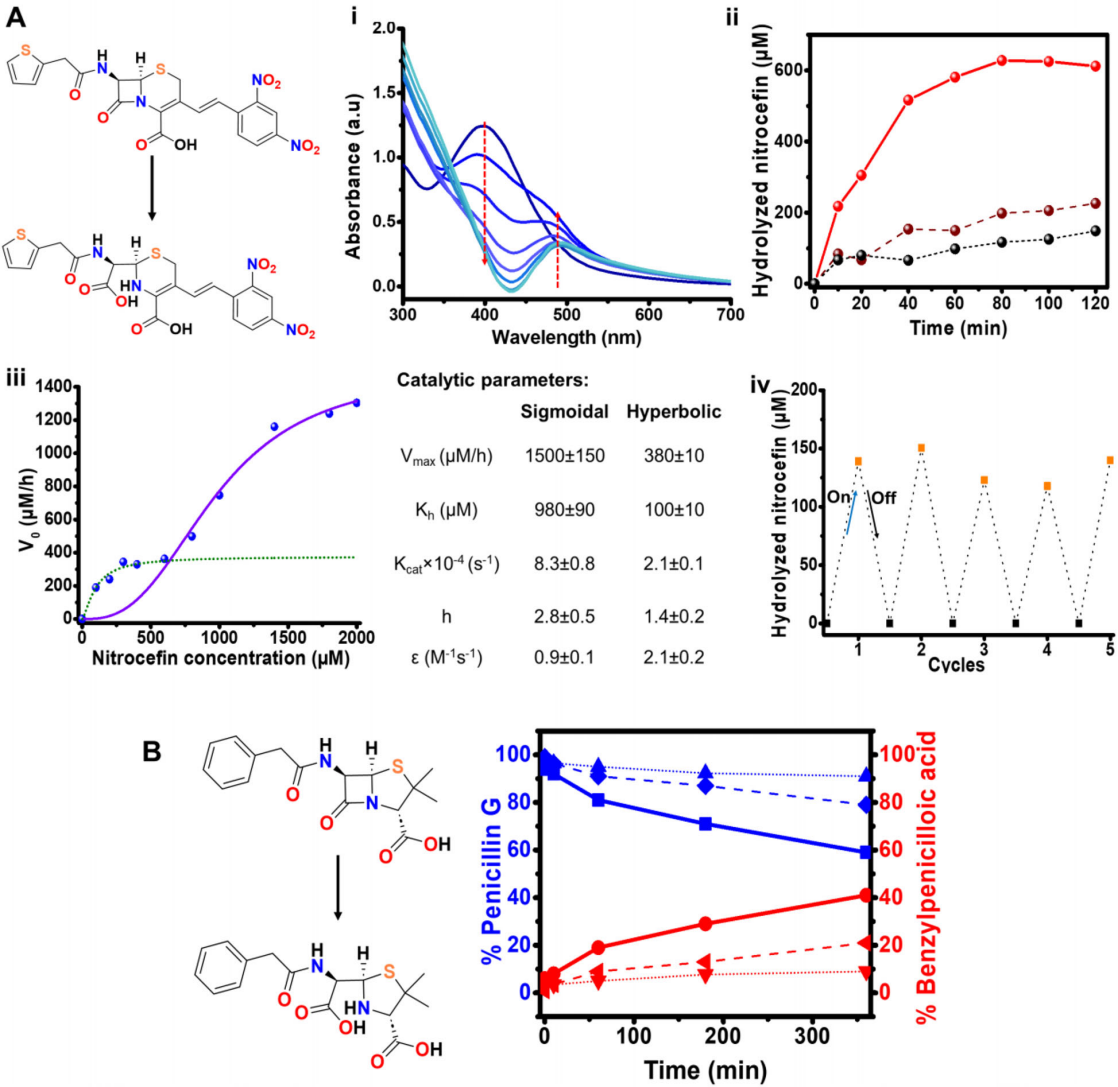
β‐lactam hydrolysis catalyzed by photo‐switched PFK/**MCH** amyloid fibrils. (A) Nitrocefin hydrolysis. Reaction scheme (top). (**i**) UV–vis spectra of nitrocefin (100 µM; baseline: PFK/**MCH** co‐mixture) added to a PFK/**MCH** mixture (1:2 mole ratio) under white light illumination (wavelength 380–750 nm, power 70 mW cm^−2^) applied for 0–80 min. The arrows indicate a decrease in the absorbance at 400 nm (nitrocefin) and an increase at 492 nm (hydrolyzed nitrocefin). (ii) Hydrolyzed nitrocefin concentrations at different times. PFK/**MCH** under white light illumination (red circles), PFK/**MCH** in dark (black circles), PFK under white light illumination (brown circles). (iii) *V_0_* vs nitrocefin concentration plot. Violet solid line: a sigmoidal fit employing all nitrocefin concentrations. Green dot line: a hyperbolic fit utilizing the low nitrocefin concentrations. The catalytic parameters for the two fits are shown in the table. (iv) Hydrolyzed nitrocefin concentrations recorded through five light on/off cycles in a PFK/**MCH** mixture (PFK and **MCH** concentrations were 0.5 and 1 mM, respectively). 0.4 mM nitrocefin was added in each cycle. (B) Hydrolysis of penicillin G. Plots of ratios of peak areas obtained from LC‐MS for penicillin G (blue lines) and hydrolyzed penicillin G (benzylpenicilloic acid, red lines) in the presence of PFK/**MCH** (1:2 mole ratios) upon white light irradiation (solid lines); PFK/**MCH** without illumination (dotted lines); no PFK/**MCH** added, with illumination (broken lines).

Importantly, the graph in Figure 6A‐ii, depicting the hydrolyzed nitrocefin (calculated from 492 nm:400 nm absorbance peak ratio) vs time underscores the photoinduced catalytic process, as experimentally significant nitrocefin hydrolysis was recorded only upon light irradiation (Figure 6A‐ii; red solid line). Indeed, the absence of catalytic activity in dark (Figure 6A‐ii; black dashed curve) further confirms that the optically switched PFK/**SP** amyloid fibrils constituted the catalytic agents. Experiments testing different PFK:**MCH** ratios in Figure S8 confirm that the photoinduced catalytic effect was linked to the formation of PFK/**SP** fibrils. Moreover, a control experiment at pH 3.3 using glycine‐HCl buffer indicates almost no degradation of nitrocefin (Figure S9), further corroborating the catalytic nature of the light‐mediated PFK/**SP** amyloid fibrils.

We calculated the catalytic parameters pertaining to the light‐mediated PFK/**SP** (1:2 mole ratio) fibrils through recording nitrocefin catalysis at different nitrocefin concentrations (Figure 6A‐iii; experimental data are presented in Figures S10 and S11). Notably, the calculated initial reaction rate (*V_0_*) can be fitted using the Hill equation according to two models, e.g., sigmoidal fit (violet solid line, which include all nitrocefin concentrations) and a hyperbolic fit (green dot line, considering only low nitrocefin concentrations). Notably, the appearance of a sigmoidal curve indicates cooperative, allosteric behavior of the PFK/**SP** fibril catalyst, yielding a high Hill constant (*h* = 2.8 ± 0.5). Such an allosteric nature mirrors the behavior of enzyme‐substrate reaction kinetics [61, 62], and this reaction mode was also observed for the previously reported salt‐induced PFK amyloid catalysis [29]. Notably, the allosteric (sigmoidal) model yields turnover number, maximal rate, and catalytic efficiency (Table in Figure 6A‐iii) that are greater than the previously reported salt‐induced catalytic PFK fibrils [29]. The enhanced catalytic efficiency is likely related to the formation of aligned fibril organization, facilitating abundant display of catalytic sites at the fibrils’ surfaces. Indeed, randomly distributed PFK/**SP_c_** amyloid fibrils (e.g., Figure 4A), exhibited significantly lower cataltyic activity compared to the photo‐induced aligned fibril system (Figure S12).

Figure 6A‐iv demonstrates that optical switching of PFK/**SP** fibril‐mediated nitrocefin catalysis could be carried out over several cycles. In the experiment presented in Figure 6A‐iv, a PFK/**MCH** solution (1:2 mole ratio) was subjected to consecutive light/dark application and nitrocefin addition (0.4 mM). Indeed, the graph in Figure 6A‐iv, depicting the hydrolyzed nitrocefin concentrations (calculated from 492 nm:400 nm absorbance peak ratios), recorded in the cycling experiments (Figure S13), reveals retention of the catalytic activity. Photoinduced PFK/**MCH** amyloid formation and nitrocefin hydrolysis also facilitated spatial patterning (Figures S14 and S15), taking advantage of the visible transformation associated with both amyloid fibril assembly and nitrocefin hydrolysis.

The optically switched PFK/**SP** amyloid fibrils catalyzed other ß‐lactam antibiotics (Figure 6B). In the experiments, we monitored hydrolysis of penicillin G, the most widely used therapeutic antibiotic [63], upon incubation with PFK/**MCH** (1:2 mole ratio) with or without illumination. The relative abundance of the parent penicillin G and its hydrolyzed product benzylpenicilloic acid were monitored by liquid chromatography‐mass spectrometry (LC‐MS) (the LC‐MS chromatograms of penicillin and benzylpenicilloic acid are presented in Figure S16). Like the nitrocefin hydrolysis experiments in Figure 6A, the graph presented in Figure 6B underscores pronounced hydrolysis of penicillin G in the presence of the photoinduced amyloid fibrils (Figure 6B, solid lines), while much lesser β‐lactam hydrolysis occurred without illumination (dotted lines). Furthermore, a solution comprising penicillin G without PFK/**SP** fibrils under illumination displayed only little hydrolysis (broken lines).

## 3 Conclusions

We report an optically switched hierarchical assembly of catalytic amyloids. The system features a unique light‐triggered assembly of the amyloidogenic peptide PFK and merocyanine photo switch (**MCH**), producing amyloid fibrils that catalyse β‐lactam hydrolysis. Fibril assembly was intimately linked to the light‐induced transformation of **MCH** to the spiropyran derivative **SP** and its interactions with PFK. Specifically, π–π interactions between the spiropyran moiety of **SP** and phenyl residues of PFK were the key determinant for fibril formation, while electrostatic attraction between the negatively charged sulfonate of **SP** and lysine sidechains of PFK contributed to the assembly of aligned long fibrils. Notably, fibril formation was fully reversible as complete disassembly to the PFK monomers occurred when light irradiation was turned off. The photoinduced PFK/**SP** amyloid fibrils were catalytic, capable of hydrolyzing ß‐lactam antibiotics, pointing to the potential utilization of the PFK/**MCH** photo switch for the removal of β‐lactam antibiotic pollutants in water.

Overall, this study presents several novel conceptual advances, including *light induced amyloid formation* – a reversible assembly of amyloid fibrils demonstrated solely by using an externally added molecule (the photoacid), which attenuates the electrostatic repulsion between individual peptides through its light induced transformation; *regulated reversible amyloid formation –* utilizing the photoacids as a “reversibility switch” furnishing a new, elegant means for achieving reversibility in amyloid fibril assembly; *Photoinduced long‐range amyloid assembly (fibril alignment)*, induced simply upon light irradiation, is unique and has not been previously reported, and *Photo‐regulated amyloid functionality (catalysis) –* demonstrating regulation (on/off) catalytic activity of amyloids through light provides a potentially powerful means of tuning amyloid functions.

In conclusion, the distinctive and simple light‐induced amyloid catalyst design involves merely co‐mixing an amyloidogenic peptide with a small molecule photo‐switch, allowing light irradiation to induce both fibrillation and catalytic activity. This intriguing concept allows spatiotemporal regulation of amyloid formation and catalysis and may have contributed to prebiotic evolutionary processes.

## 4 Experimental Section

### 4.1 Materials

The peptide Pro‐Lys‐(Phe‐Lys)5‐Pro (M_w_ of 1717.14; PFK) was custom‐synthesized and purified to 95% by HPLC and supplied as lyophilized powder by GL Biochem (China). 2,3,3‐trimethylindolenine and 1,3,3‐trimethylindolinobenzopyrylospiran (**SP_c_**) were purchased from Aron Chemical (China). Triethylamine, 1,3‐propanesultone, and 2‐hydroxylbenzaldehyde were purchased from Merck (Germany), Thermo Scientific‐Alfa Aesar (USA), and Chem‐Impex (USA), respectively. Amytracker‐680 was supplied by Ebba biotech (Sweden). Nitrocefin [3‐(2,4‐dinitrostyryl)‐(6 R,7 R)‐7‐(2‐thienylacetamido)‐ceph‐3‐em‐4‐carboxylic acid] was purchased from Holland‐Moran (Israel). Penicillin G sodium salt (96%) was purchased from Sigma–Aldrich (Germany). Dimethyl sulfoxide (DMSO, 99.9% purity, analytical grade) and ethanol (99%) were purchased from Fischer Chemicals (Switzerland) and Romical (Israel), respectively. Diethyl ether (CP) and tetrahydrofuran (AR) were purchased from Bio‐Lab Ltd (Israel). Ultrapure water (18.2 mΩ.cm at 25°C) by Synergy UV water purification system was used.

### 4.2 Synthesis

#### 4.2.1 Synthesis of (E)‐3‐(2‐(2‐hydroxystyryl)‐3,3‐dimethyl‐3H‐indol‐1‐ium‐1‐yl)propane‐1‐Sulfonate (MCH)

**MCH** was synthesized according to previously published protocols [64]. Briefly, 2,3,3‐trimethylindolenine (1.65 g, 10 mmol) and 1,3‐propanesultone (1.26 g, 10 mmol) were added to a 50 mL round‐bottom flask and stirred at 90°C for 4 h. A purple solid obtained as a precipitate was collected by filtration, washed with cold diethyl ether (5 × 10 mL^2^), and finally dried under vacuum. The obtained solid (2.1 g, 7.2 mmol) and 2‐hydroxylbenzaldehyde (0.96 g, 7.8 mmol) were taken in a 50 mL round‐bottom flask, and 30 mL anhydrous ethanol was added. The mixture was stirred overnight under reflux conditions. An orange solid obtained as a precipitate was collected by filtration, washed with cold ethanol (3 × 10 mL^2^), and finally dried under vacuum in the dark. Yield: 2.06 g, 72%.

#### 4.2.2 Synthesis of 3‐(triethylammonio)propane‐1‐sulfonate (TEAPS)

1,3‐propanesultone (1.0 g, 8.19 mmol) and triethylamine (50 equiv.) in 5 mL tetrahydrofuran were stirred at 35°C for 6 h. The white precipitate obtained was collected by filtration, washed with cold diethyl ether (3 × 10 mL), and finally dried under vacuum. Yield: 972 mg, 66%.

### 4.3 Sample Preparations

#### 4.3.1 Preparation of PFK and PFK/MCH Solutions, and PFK/SP Fibrils

0.5 mM PFK solution was prepared by dissolving PFK lyophilized powder (0.9 mg) in 10% DMSO in water (1 mL). pH of the solution was 5.28. To prepare PFK/**MCH** (1:2) solution, at first, 1.0 mM **MCH** solution was prepared by dissolving **MCH** (2 mg) in 10% DMSO in water (5 mL) under dark conditions and used for further experiments. Then, 1.0 mM **MCH** solution (1.05 mL) was added to 0.9 mg PFK lyophilized powder in dark to obtain PFK/**MCH** (1:2) solution. pH of the solution was 5.22. PFK/**MCH** solutions were then irradiated with WLED (380–750 nm range, power of 70 mW cm^−2^) for 5–10 min to obtain PFK/**SP** fibrils. The alignment of the fibrils was dependent on the initial ratio of PFK/**MCH** and irradiation time.

#### 4.3.2 Preparation of SP_c_ Solution and PFK/SP_c_ Fibrils

At first, 1.0 mM 1,3,3‐Trimethylindolinobenzopyrylospiran (**SP_c_**) solution was prepared by dissolving **SP_c_** (1.4 mg) in 10% DMSO in water. Then, 1.0 mM **SP_c_** solution (1.05 mL) was added to the lyophilized powder of PFK (0.9 mg) and kept for 20 min to obtain PFK/**SP_c_** (1:2) fibrils.

#### 4.3.3 Preparation of TEAPS and PFK/TEAPS Solutions

1.0 mM **TEAPS** solution was first prepared by dissolving Et_3_NC_3_SO_3_ (1.1 mg) in 10% DMSO in water. Then, 1.0 mM **TEAPS** solution (1.05 mL) was added to the lyophilized powder of PFK (0.9 mg) to obtain PFK/**TEAPS** (1:2) solution.

#### 4.3.4 Preparation of Nitrocefin and Penicillin G Solutions for Hydrolysis

Nitrocefin (2 mg) was dissolved in acetonitrile (0.2 mL) to obtain a 20 mM solution. 0.1 mL of a 20 mM nitrocefin solution was added to 1 mL of PFK/**MCH** solution for hydrolysis of nitrocefin (2 mM) in both dark and light conditions. Penicillin G sodium salt (1.4 mg) was dissolved in water (0.2 mL) to obtain a 20 mM solution. 0.1 mL of 20 mM penicillin G solution was immediately added to 1 mL of PFK/**MCH** solution for hydrolysis of penicillin G (2 mM) in both dark and light conditions.

#### 4.3.5 Preparation of Samples for Cryogenic Transmission Electron Microscopy (cryo‐TEM)

For the Cryo‐TEM imaging of the PFK/**MCH** sample in the dark, a 2.5 µL drop of the solution was applied to the TEM grid under dark conditions and blotted. The blotted samples were immediately plunged into liquid ethane at its freezing point (−181°C). For cryo‐TEM imaging of the light‐irradiated samples, PFK/**MCH** solutions were irradiated with light, then immediately transferred to the TEM grid (2.5 µL) under light, blotted, and plunged into liquid ethane at its freezing point (−181°C).

### 4.4 Instrumentation

#### 4.4.1 Cryogenic Transmission Electron Microscopy (cryo‐TEM)

Vitrified specimens were prepared on a glow‐discharged copper grid coated with a perforated lacey carbon 300 mesh (Ted Pella Inc.). A typically 2.5µl drop from the solution was applied to the grid and blotted with a filter paper to form a thin liquid film of solution. The blotted samples were immediately plunged into liquid ethane at its freezing point (−181°C). The procedure was performed automatically in the Plunger (Lieca EM GP2). The vitrified specimens were then transferred into liquid nitrogen for storage. The samples were studied using a Talos F200C TEM Thermo Fisher Scientific, at 200kV maintained at liquid nitrogen temperature; and images were recorded on a Ceta 16M camera (4k × 4k CMOS sensor) Thermo Fisher Scientific at low dose conditions, to minimize electron beam radiation damage.

#### 4.4.2 UV–vis and Fluorescence Spectroscopy

UV–vis spectra were recorded in a Thermo Scientific Evolution 220 UV spectrophotometer with a quartz cuvette having a path length of 0.1 cm. For the nitrocefin hydrolysis experiments, UV–vis spectra were recorded in a Biotek Synergy H1 plate reader by placing 400 µL of the diluted (20 times) solutions from the reaction mixture in a 96‐well plate. Fluorescence spectra were recorded in a Horiba Fluorolog fluorometer. For Amytracker‐680 fluorescence measurements, the samples were excited at 580 nm wavelength. For **MCH** fluorescence without and with PFK, the excitation wavelength was 420 nm. To measure **SP** fluorescence, for **SP** alone or in PFK/**SP** fibrils, the samples were excited at 300 nm wavelength. The PFK autofluorescence in the presence of **SP**, **SP_c_**, or **TEAPS** was recorded upon exciting at 240 nm wavelength.

#### 4.4.3 Attenuated Total Reflection Infrared (ATR‐IR) Spectroscopy

ATR‐IR spectra of the sample solutions were monitored using a NICOLET 6700 FTIR spectrometer equipped with an attenuated total reflectance (ATR) system featuring a diamond crystal and a DTGS detector (Thermo Fisher Scientific, MA, USA).

#### 4.4.4 Circular dichroism (CD) spectroscopy

CD spectra were recorded in a range of 190–260 nm on a Jasco J‐715 spectropolarimeter (Tokyo, Japan), with 0.01 cm quartz cuvettes. The volume of the samples was 40 µL.

#### 4.4.5 Liquid Chromatography‐Mass Spectrometry (LC‐MS)

Aliquots (10 µL) from the hydrolysis reaction of Penicillin G in LC‐MS grade water and in the presence of PFK/**MCH** solutions in dark and light conditions were collected at 0, 10, 60, 180, and 360 min intervals. The collected samples were immediately mixed with 90 µL of LC‐MS grade methanol to quench further reaction. For the LC‐MS analysis, the samples were centrifuged, moved to the LC‐vials, and injected subsequently. A Waters ACQUITY UPLC I‐Class PLUS System equipped with an Acquity UPLC BEH C18 column (1.7 µm, 2.1 × 50 mm^2^) and coupled with a high‐resolution Q‐Exactive Plus mass spectrometer (Thermo Scientific, MA, USA) with an electrospray ionization source was used in the analysis.

#### 4.4.6 ζ Potential Measurements

ζ potentials were measured using Zetasizer Nano ZS instrument (Malvern, Worcestershire, UK). Samples were placed inside a folded capillary cell (part number DTS1070) equipped with copper electrodes, and the zeta potentials were recorded.

#### 4.4.7 NMR Spectroscopy

NMR spectra were recorded in a Bruker AVANCE III‐500 NMR spectrometer. The NMR samples for PFK (0.5 mM), **MCH** (1.0 mM), and PFK (0.5 mM)/**MCH** (1.0 mM) were prepared in D_2_O. The NMR samples for **SP_c_** (1.0 mM) and PFK (0.5 mM)/**SP_c_** (1.0 mM) were prepared in 10% DMSO‐D_6_ in D_2_O. For the NMR spectra measurement under light, an optical fiber with 405 nm light source was put inside the NMR solution and then the spectra were recorded without spinning.

### 4.5 Calculations

#### 4.5.1 Calculation for Hydrolyzed Nitrocefin Concentrations

The concentration of hydrolyzed nitrocefin ($C_{HNC}^{t}$) at a time *t* was calculated using the following formula‐

$$C_{HNC}^{t}=C_{NC}^{0}-\frac{C_{NC}^{0}}{\frac{A_{492}^{t}}{A_{400}^{t}}\frac{\varepsilon \mathrm{NC}}{\varepsilon \mathrm{HNC}}+1}$$

where, $C_{NC}^{0}$ is the initial nitrocefin concentration; $\frac{A_{492}^{t}}{A_{400}^{t}}$ is the absorbance ratio at 492 nm (corresponding to hydrolyzed nitrocefin) and 400 nm (corresponding to non‐hydrolyzed nitrocefin) at time *t*; εNC and εHNC are molar absorptivity of nirocefin (∼11 500 M^−1^cm^−1^) and hydrolyzed nitrocefin (∼17420 M^−1^cm^−1^) [65].

#### 4.5.2 Kinetic Analysis

Initial rates (*V_0_*) were first calculated from the slopes of the initial reaction time (0–10 min) at different nitrocefin concentrations. Then the initial rates (*V_0_*) were plotted against different nitrocefin concentrations using the Hill equation, i.e.,

$$V=\frac{V_{max}{\left[s\right]}^{h}}{k_{h}^{h}+{\left[s\right]}^{h}}$$

where, *V_max_* is the maximum reaction rate, *h* is the Hill coefficient *k_h_* represents half‐maximal concentration constant, and [*s*] is the substrate, i.e., nitrocefin concentration.

#### 4.5.3 Calculation of Turnover Number and Catalytic Efficiency

Turnover number (*k_cat_*) was calculated using the following equation‐

$$k_{cat}=\frac{V_{max}}{\left[Cat\right]}$$

where [*Cat*] is the catalyst i.e., PFK concentration.

Catalytic efficiency (*ε*) was calculated using the following equation‐

$$\varepsilon =\frac{k_{cat}}{k_{h}}$$

## Author Contributions

**Shubhra Kanti Bhaumik**: investigation, writing – original draft, methodology, formal analysis. **Sisira Mambram Kunnath**: validation, formal analysis. **Elad Arad**: investigation, methodology. **Einat Nativ‐Roth**: formal analysis, data curation. **Arunavo Chatterjee**: methodology, validation. **Albert Batushansky**: data curation, formal analysis. **Nitzan Shauloff**: investigation, validation, methodology. **Hanna Rapaport**: data curation. **Raz Jelinek**: conceptualization, funding acquisition, writing – review and editing, project administration, supervision, resources.

## Conflicts of Interest

The authors declare no conflicts of interest.

## Supporting information




**Supporting File**: smll75302‐sup‐0001‐SuppMat.docx.

## Data Availability

The data that supports the findings of this study are available in the Supporting Information of this article.
