# Supplementary material for: Photo‐Switched Catalytic Amyloids
Source: Small. 2026 Aug 18;22(60):e75302. doi: 10.1002/smll.75302 (PMC13711468; doi:10.1002/smll.75302)
Supplement: Supplementary file 1 [file SMLL-22-e75302-s001.docx]

Supporting Information

Photo-Switched Catalytic Amyloids

Shubhra Kanti Bhaumik, Sisira Mambram Kunnath, Elad Arad, Einat Nativ-Roth, Arunavo Chatterjee, Albert Batushansky, Nitzan Shauloff, Hanna Rapaport, and Raz Jelinek*

Supporting Figures

1. Polarized optical microscopic images

**Figure S1.** Polarized optical microscopic (POM) images of the nematic phase-like domain of the PFK/**SP** fibrils under light. **A.** Brightfield image. **B.** Image under cross-polarizers showing birefringent nematic domain. Scale bar: 5 µm. *Polarized optical microscopy imaging:* 5 µL of solution containing PFK/**SP** fibrils formed under light was placed under a ZEISS Axio Imager 2 microscope connected with a monitor to record the images in brightfield and under cross-polarizers.

2. Cryogenic TEM of PFK fibrils

Figure S2. A. Cryogenic TEM image of PFK amyloid fibrils upon incubation with 0.2 M KCl. B. Fibril width distribution of PFK, determined from the TEM image.

**3. Spectroscopic data for PFK/MCH co-mixture or PFK/SP fibrils**

**Figure S3.** ATR-IR spectra of PFK/**MCH** in the dark, PFK/**SP** under light irradiation, and PFK/**MCH** in the dark after 30 min of light irradiation in D_2_O.

**Figure S4.** Fluorescence spectra of **MCH** in water/DMSO (9:1).

**4. Spectroscopic data for PFK/SP_c_ fibrils**

**Figure S5.** **A.** CD spectra of PFK, **SP_c_**, and PFK/**SP_c_** in water/HFIP (9:1). HFIP was added as a co-solvent to record the spectra. **B.** ATR-IR spectra of PFK, **SP_c_**, and PFK/**SP_c_** in D_2_O/CD_3_OD (9:1).

**Figure S6.** ^1^H NMR spectra of **SP_c_** and PFK/**SP_c_** in 10% DMSO-D_6_ in D_2_O (aromatic region: 5.5-8 ppm).

**5. Spectroscopic data for PFK/TEAPS co-mixture**

**Figure S7.** CD spectra of PFK and PFK/**TEAPS** in water.

**6. Nitrocefin hydrolysis**

**Figure S8.** UV-vis spectral changes of nitrocefin (20-times diluted from 2 mM, baseline: PFK-**MCH**) at different times in the presence of **A.** PFK (0.5 mM)/**SP** (0.5 mM), **B.** PFK (0.5 mM)/**SP** (0.25 mM), **C.** PFK (0.5 mM)/**SP** (0.1 mM), **D.** PFK (0.5 mM), **E.** **SP** under light, and **F.** PFK (0.5 mM)/**MCH** (1.0 mM) under dark conditions. **G.** Plots of hydrolyzed nitrocefin (calculated from A_492_/A_400_) at different times in the presence of PFK/**MCH** under dark and PFK/**SP** with different PFK/**SP** ratios in light conditions.

**Figure S9.** UV-vis spectral changes of nitrocefin (20-times diluted from 2 mM) at different times in glycine-HCl buffer (pH 3.3, 1 mM).

**Figure S10.** (**A**-**I**) UV-vis spectral changes of nitrocefin (20-times diluted, baseline: PFK/**MCH**) at different times for different nitrocefin concentrations using PFK/**SP** (1:2 mole ratio) under light.

**Figure S11.** Hydrolyzed nitrocefin (calculated from 492 nm:400 nm absorbance peak ratio) vs time at different nitrocefin concentrations using PFK/**SP** fibrils under light.

**Figure S12.** Nitrocefin hydrolysis by PFK/**SP_c_** (1:2 mole ratio) amyloid fibrils. **A.** UV-vis spectral changes of nitrocefin (20-times diluted, baseline: PFK/**SP_c_**) at different times. **B.** Comparison plot showing of hydrolyzed nitrocefin vs time using photo induced PFK/**SP** amyloid fibrils and PFK/**SP_c_** fibrils.

**Figure S13.** (**A**-**E**) UV-vis spectral changes of nitrocefin (20 µM, diluted from 0.4 mM) in the presence of PFK (0.5 mM)/**SP** (1.0 mM) under light for 5 consecutive cycles. Baseline was PFK/**MCH** for first cycle and for next cycles the baseline was the same PFK/**MCH** solution containing hydrolyzed nitrocefin product.

**Figure S14.** Spatially regulated patterning via photoinduced fibrillation of PFK/**SP**. **A.** The experimental setup. Light can penetrate and irradiate a thin PFK/**SP** film through a prefabricated hole. **B.** Images of the petri dish containing PFK-**MCH** thin layer before and after irradiation The indicated region is lighter in color due to fibril formation.

**Figure S15.** Visual patterning due to light-induced catalytic hydrolysis of nitrocefin by PFK/**SP** fibrils. Images of solutions of PFK-**MCH**-nitrocefin (**i**), PFK-**MCH** (**ii**), PFK-nitrocefin (**iii**), and **MCH**-nitrocefin (iv) in the wells **A.** before irradiation and **B.** after irradiation. The color of the solution containing PFK-**MCH**-nitrocefin changed to reddish-brown due to nitrocefin hydrolysis.

**7. Penicillin G hydrolysis**

**Figure S16.** LC-MS analysis of **Penicillin G** and hydrolyzed **Penicillin G** (benzylpenicilloic acid) by catalytic degradation employing light-induced amyloid-like fibrils of PFK/**MCH** (1:2 molar ratio). **A.** LC Gradient protocol for **Penicillin G** and hydrolyzed **Penicillin G** (benzylpenicilloic acid). **B.** LC-MS chromatogram of **Penicillin G**. In the inset, the detected mass spectra of **Penicillin G** and the theoretically calculated values are shown. **C**. LC-MS chromatogram of hydrolyzed **Penicillin G** (benzylpenicilloic acid). In the inset, the detected mass spectra of hydrolyzed **Penicillin G** (benzylpenicilloic acid) and the theoretically calculated values are shown. The column temperature was maintained at 40 °C. A constant flow rate of 0.4 mL/min was applied throughout the experiment, with a total runtime of 15 minutes. Mobile phase (MP) A consisted of water with 0.1% formic acid, and MP B consisted of acetonitrile with 0.1% formic acid. The solvents (water, formic acid, acetonitrile) were used in LC-MS grade.

**8. Additional data: Synthetic route, NMR spectra, and NMR data for TEAPS**

**A.** Chemical reaction scheme for the synthesis of **TEPS** from 1,3-propane sultone and triethyl amine in THF. **B.** ^1^H NMR spectra of **TEPS** in D_2_O. ^1^H NMR (500 MHz, D_2_O): δ (ppm) =3.30-3.37 (m, 8H), 2.99 (t, J = 10 Hz, 2H), 2.14 (quintet, J = 10 Hz, 2H), 1.29 (t, J = 10 Hz, 9H).
